# A novel scaled-gamma-tanh (SGT) activation function in 3D CNN applied for MRI classification

**DOI:** 10.1038/s41598-022-19020-y

**Published:** 2022-09-02

**Authors:** Bijen Khagi, Goo-Rak Kwon

**Affiliations:** 1 Information and Communication Engineering, Chosun University, Gwangju, 61452 South Korea; 2 Gwangju Alzheimer’s Disease and Related Dementia Cohort Research Center, Gwangju, South Korea

**Keywords:** Computational biology and bioinformatics, Neuroscience, Health care, Medical research, Engineering, Nanoscience and technology

## Abstract

Activation functions in the neural network are responsible for ‘firing’ the nodes in it. In a deep neural network they ‘activate’ the features to reduce feature redundancy and learn the complex pattern by adding non-linearity in the network to learn task-specific goals. In this paper, we propose a simple and interesting activation function based on the combination of scaled gamma correction and hyperbolic tangent function, which we call Scaled Gamma Tanh (SGT) activation. The proposed activation function is applied in two steps, first is the calculation of gamma version as *y* = *f*(*x*) = *ax*^*α*^ for *x* < *0* and *y* = *f*(*x*) = *bx*^*β*^ for *x* ≥ *0*, second is obtaining the squashed value as *z* = tanh(*y*). The variables *a* and *b* are user-defined constant values whereas $\alpha$ and $\beta$ are channel-based learnable parameters. We analyzed the behavior of the proposed SGT activation function against other popular activation functions like ReLU, Leaky-ReLU, and tanh along with their role to confront vanishing/exploding gradient problems. For this, we implemented the SGT activation functions in a 3D Convolutional neural network (CNN) for the classification of magnetic resonance imaging (MRIs). More importantly to support our proposed idea we have presented a thorough analysis via histogram of inputs and outputs in activation layers along with weights/bias plot and t-SNE (t-Distributed Stochastic Neighbor Embedding) projection of fully connected layer for the trained CNN models. Our results in MRI classification show SGT outperforms standard ReLU and tanh activation in all cases i.e., final validation accuracy, final validation loss, test accuracy, Cohen’s kappa score, and Precision.

## Introduction

An activation function is primarily used in DNN for two purposes, first to add non-linearity in the whole system to learn complex patterns and second to normalize or threshold the output of each layer to reduce the computational burden^1,2^. Here, for a CNN, if only linear activation *f*(*x*) = *wx* + *b* is used, then stacking multiple functions of f(x) produces only a single degree output noting that the convolution layer itself is also a linear operation layer^3^. Aside output values can monotonically explode to a maximal or minimal level causing difficulty in training to reach convergence. Hence, the learned polynomial expression should be in order greater than 1 to learn complex patterns due to multi-dimension features^4,5^ i.e., the decision boundary needs to be non-linear. For this, the activation functions need to be chosen properly in deep networks as it has significant effects on the training dynamics and required task performance^4,6,7^.

Traditionally neural networks implementing Multilayer Perceptron (MLP) used sigmoid function or tanh as a non-linear operator in its neuron or node^8–11^. Later with emerging complexity in DNN, many other activation functions based on the non-linear operation were proposed. However, most of them were highly complex and designed for a very deep network for their high-level abstract representation in natural image datasets like ImageNet^12–14^. It made the network more complex to understand its working mechanism and feature extraction process^15^. Thus, still simpler non-linear rectifiers like ReLU^16^ and its variants Leaky-ReLU^17^ are the most popular ones along with other Parametric ReLU (P-ReLU)^18^, GELU^19^, ELU^20^, SELU^15^ being occasionally used in DNN like CNN^8,21–23^. Zhang et al.^3^ in their work for CNN improvement have supported the use of non-linear transformation for convolution layer and FCL. The addition of asymmetric kernel approximation has improved the classification task and generalization ability. Likewise, Hayou et al.^1^ studied the impact of activation functions on DNN and concluded that inappropriate selection can lead to the loss of information of the input during forward-propagation and the exponential vanishing/exploding of gradients during back-propagation. Recently, Dubey et al.^2^ performed a comprehensive survey on performance analysis in deep learning, to understand the behavior of non-linear transformation of activation function. ReLU defined as f(x) = max (0, x) completely blocks the negative input for positive gradient flow whereas its other variants allow a computed flow of negative input for small negative gradients loss. Although the vanishing gradient problem was solved with positive gradients loss in ReLU, it gave rise to another similar problem called ‘dying ReLU’, which is encountered if higher negative input keeps on prevailing at the cost of sparsity. Later these problems were solved using Leaky-ReLU and P-ReLU^16,18^ with non-zero activation for negative inputs as *f*(*x*) = *αx*, where *α* is a constant scalar or a learnable parameter. However, in the case of medical image classification like MRI and Positron Emission Tomography (PET), ReLU and Leaky-ReLU are still the dominant ones due to their simplicity and training images being in greyscale format. Recent works in MRI classification using DNN include designing robust and better architecture, ensemble models along with clinical features, and experiments to apply new learning and optimization algorithms^24–28^. While very few works have been done in designing novel activation functions specifically to MRI, as most researchers use the existing activation methods^29–31^. Hosseini-Asl et al.^24^ used Sigmoid and ReLU function to design deeply supervised and adaptable 3D CNN (DSA-3D-CNN) trained on structural MRI (sMRI) images, for the prediction of Alzheimer’s disease (AD) vs. mild cognitive impairment (MCI) vs. controlled normal (CN) task. Payan et al.^25^ proposed sparse auto-encoder (SAE) patch-based 3D CNN using sigmoid activation function to classify MRI scans. Similarly, Oh et al.^26^ performed fivefold cross-validations (CV) using convolutional auto-encoder (CAE) based volumetric CNN with ReLU as the activation function for AD vs. NC classification along with supervised transfer learning for sMCI vs. pMCI classification. Gupta et al.^32^ used CNN with sigmoid activation function to classify MRI into 3 classes with transferred features learned from natural images using autoencoder. E.Goceri^28^ proposed Sobolev gradient-based optimization for 3D-CNN, results for MRI classification accuracy were reported higher with Leaky-ReLU in comparison to sigmoid and ReLU. Recently Huang et al.^29^ implemented a combination of GELU and ReLU in their DNN model for brain tumor image classification and achieved a 95.49% success rate.

**Figure 1:**
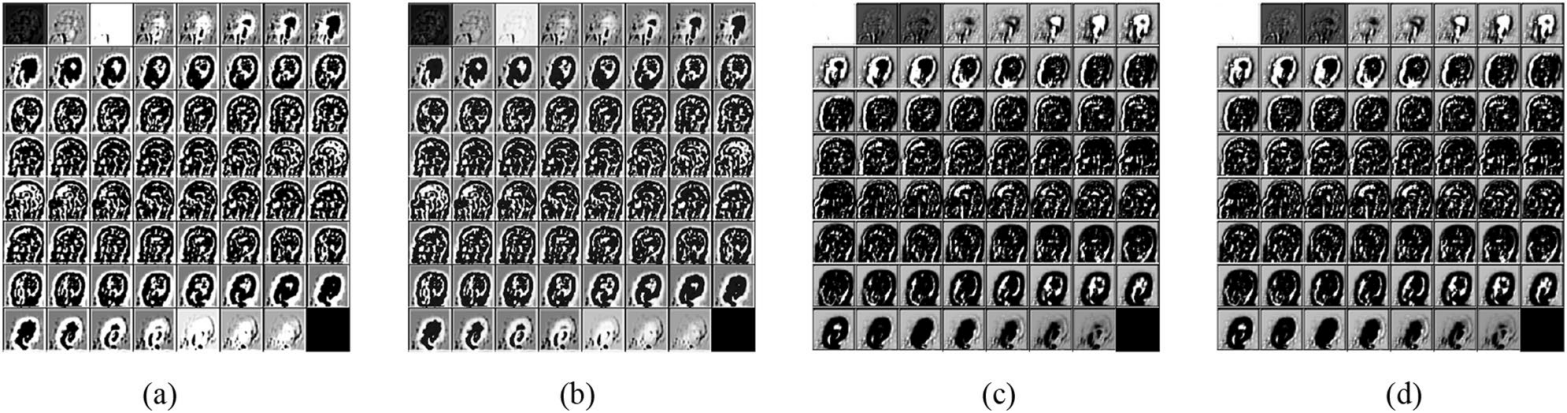
Comparison of activation using different functions for a sample MRI observed in 2nd activation layer (22nd of 64 channels) corresponding to 63 × 63 × 63 image as a montage from (**a**) Input (**b**) Output using SGT (**c**) Output using ReLU (**d**) Output using Leaky-ReLU. It can be observed that the output from the SGT, Fig. 1b), has well preserved the feature attribute present in the first three and last few slices in comparison to ReLU (**c**) and Leaky ReLU (**d**) where (**a**) is the input feature matrix.

Generally, Gamma correction (*f*(*x*) = *x*^*γ*^)^33^ is about contrast enhancement and non-monotonically intensity mapping to new values, depending on the exponent *γ* for the input *x*. In deep learning scenarios, Gamma correction is mostly used to produce augmented images (with defined *γ* values like *γ* = 0.5,1.5,2, etc.) for increasing training material^34–36^. This idea seems helpful to increase the training result by producing multiple versions of gamma-corrected images using different values of *γ* in *f*(*x*) = *x*^*γ*^. However, it should also be noted that several image’s quality might deteriorate due to the unmatched version of gamma. With the higher value of *γ*, we can wash out the image whereas with the lower value of *γ* we might lose the important pixel information. Hence ‘*γ’* should be a ‘versatile’ constant or technically a learnable parameter as per channels rather than a ‘fixed’ constant. Hence our idea is to select an appropriate gamma value for each image, or more specifically for all the images (or their features) obtained from all the channels output after Batch normalization (BN). Hence our method is not to increase the number of augmented images rather to find appropriate values of gamma for each filter output and bring non-linearity in the model at the same time, without increasing the number of training samples which works as an activation function (please see Fig. 1).

In this work we contributed as follows:

- A novel activation function is proposed with the stepwise combination of gamma correction technique and hyperbolic tangent function. Although zero-centered symmetric functions like Sigmoid, and tanh is desirable for activation function for un-skewed gradients however, those functions proved to be not worthy due to the vanishing gradient problem^2,37^. The best proven recent activation functions are mostly non-symmetrical around zero, hence we are also developing a non-symmetric function. For the application of our proposed idea, we have implemented the proposed SGT activation technique for MRI classification using our previously used architecture^38^ with a reduced number of fully connected layers.
- Various experimental analyses are introduced to support our findings. Since each activation layer is preceded by BN layer, the idea is to distribute histogram with saturation at low and high intensities of input data, which was originally mean centered at zero with unit variance. In other words, the intensity profile is dispersed from the central region to the edges. This brings higher variance in weight distribution with significant discrimination in features to support the classification (please see histogram distribution Fig. 7).

## Proposed SGT activation and training process

The proposed SGT activation is performed in two steps:

1 $${\text{Step 1}}:y = f\left( x \right) = ax^{\alpha } \;{\text{for}}\;x < 0\;{\text{and}}\;bx^{\beta } \;{\text{for}}\;x > 0$$

Here the first step is finding the gamma corrected version of input *x* as in Eq. (1). *x* is an input defined by a 4D matrix/Tensor as $X_{l}$ with each pixel/feature value $X_{n}^{b}$ for ‘*b*th*’* batch and ‘*n*th*’* filter in layer $^{\prime}l^{\prime}$. *a* and *b* are constant scaling factors that were set manually. For *n* filters, we have *n* values of learnable parameters (i.e., $\alpha {\text{ or }}\beta$) which implies that for all the different (or same)-class images belonging to the same mini-batch, the value of exponent remains the same, whereas the value of the exponents is different for the same-class images in different channels, hence are activated differently in each channel as shown in matrix representation in Eq. (2), ^ signifies operation performed in column to column element wise exponential operation.

2 $$\begin{aligned} Y_{l} = & a \cdot \left[ {\begin{array}{*{20}c} {X_{1}^{1} } & \cdots & {X_{1}^{b} } \\ \vdots & \ddots & \vdots \\ {X_{n}^{1} } & \cdots & {X_{n}^{b} } \\ \end{array} } \right]{ \wedge }\left[ {\begin{array}{*{20}c} {\alpha _{1} } \\ \vdots \\ {\alpha _{n} } \\ \end{array} } \right] = a \cdot \left[ {\begin{array}{*{20}c} {X_{1}^{{1\alpha _{1} }} } & \cdots & {X_{1}^{{b\alpha _{1} }} } \\ \vdots & \ddots & \vdots \\ {X_{n}^{{1\alpha _{n} }} } & \cdots & {X_{n}^{{b\alpha _{n} }} } \\ \end{array} } \right] = \left[ {\begin{array}{*{20}c} {Y_{1}^{1} } & \cdots & {Y_{1}^{b} } \\ \vdots & \ddots & \vdots \\ {Y_{n}^{1} } & \cdots & {Y_{n}^{b} } \\ \end{array} } \right]~\quad ({\text{for}}~X_{n}^{b} < 0~) \\ = & b \cdot \left[ {\begin{array}{*{20}c} {X_{1}^{1} } & \cdots & {X_{1}^{b} } \\ \vdots & \ddots & \vdots \\ {X_{n}^{1} } & \cdots & {X_{n}^{b} } \\ \end{array} } \right]{ \wedge }\left[ {\begin{array}{*{20}c} {\beta _{1} } \\ \vdots \\ {\beta _{n} } \\ \end{array} } \right] = b \cdot \left[ {\begin{array}{*{20}c} {X_{1}^{{1\beta _{1} }} } & \cdots & {X_{1}^{{b\beta _{1} }} } \\ \vdots & \ddots & \vdots \\ {X_{n}^{{1\beta _{n} }} } & \cdots & {X_{n}^{{b\beta _{n} }} } \\ \end{array} } \right] = \left[ {\begin{array}{*{20}c} {Y_{1}^{1} } & \cdots & {Y_{1}^{b} } \\ \vdots & \ddots & \vdots \\ {Y_{n}^{1} } & \cdots & {Y_{n}^{b} } \\ \end{array} } \right]~\;\quad ({\text{for}}~X_{n}^{b} > 0~) \\ \end{aligned}$$

where $$X_{l} = \left[ {\begin{array}{*{20}c} {X_{1}^{1} } & \cdots & {X_{1}^{b} } \\ \vdots & \ddots & \vdots \\ {X_{n}^{1} } & \cdots & {X_{n}^{b} } \\ \end{array} } \right]$$ is the input to the layer $l$.

**Figure 2:**
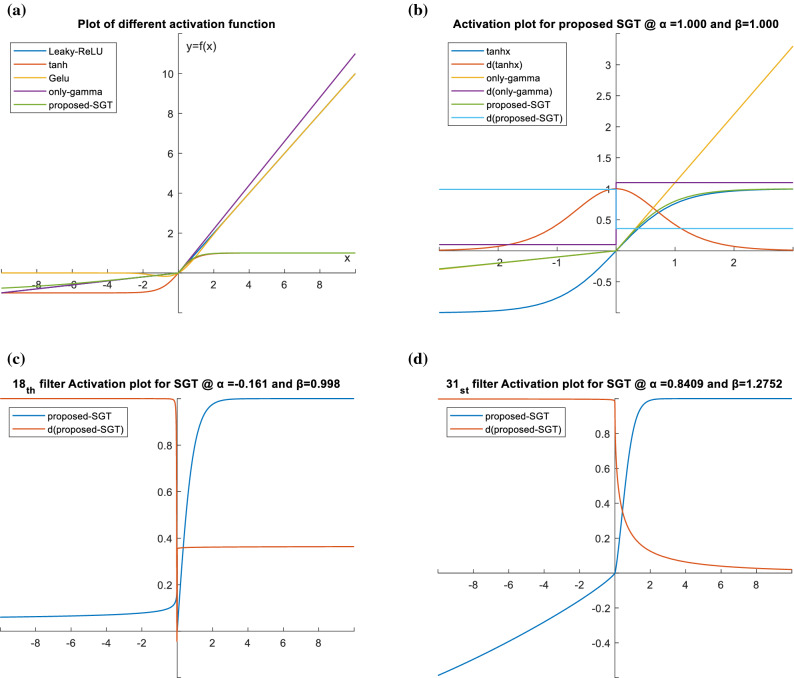
(**a**) Activation function plot for input x and f(x) along with other popular activation functions near x = 0. Please see "Appendix" for all the related equations. Please see Fig. 2_app in "Appendix" for all the related equations. (**b**) Activation (proposed-SGT) and first-order derivative (d(proposed-SGT)) plot with both exponents equal to 1 using a combination of gamma correction (‘only-gamma’) and hyperbolic tangent (‘tanh’) to illustrate the need for thresholding and squashing function. (**c**) Actual activation plot for the trained network in 18^th^ filter (out of 64) in layer 4. Here blue curve represents the SGT activation function whereas the red curve represents its first-order derivative. (**d**) Actual activation plot for the trained network in 31st filter (out of 64) in layer 4. Here blue curve represents the SGT activation function whereas the red curve represents its first-order derivative.

Here *a* and *b* are scaling constants selected manually, for our case we have selected to 0.1 and 1.1 respectively. It is done to behave slightly as a monotonic function when the exponents are equal to 1 and resemble the Leaky-ReLU function in the first step (please see Fig. 2a). Later in the second step, when passed through the hyperbolic tangent (both exponents as 1) function, the output for the positive part will resemble tanh, and for the negative part will partly resemble the Leaky-ReLU function (please see Fig. 2b). However, on changing the exponent value and sign, different activation plots can be generated as shown in Fig. 2c and d. Here it should be noted that only using step 1 for activation might explode the activated value in the positive region and can lead to vanishing gradient in the negative region (please see ‘only-gamma’ plot in Fig. 2b) which causes computational difficulty in convergence during training. Therefore, a thresholding function with non-linear and symmetric property in positive and negative axis is required, for which we have selected the tanh function. The learnable parameters α and β values work as a positive gamma corrector, hence the weight updates of value *α* and *β* are calculated from the partial derivative of Eq. (1) during backward propagation as in Eqs. (3) and (4):

3 $$\frac{{{\text{d}}l}}{{{\text{d}}\alpha }} = \mathop \sum \limits_{b} \mathop \sum \limits_{n} 0.1 \times real\left( {\log_{10} X_{b}^{n} } \right) \cdot real\left( {X_{b}^{n\alpha } } \right) \cdot \frac{{{\text{d}}l}}{{{\text{d}}z}} \,for\, X_{n}^{b} < 0$$

4 $$\frac{{{\text{d}}l}}{{{\text{d}}\beta }} = \mathop \sum \limits_{b} \mathop \sum \limits_{n} 1.1 \times real\left( {\log_{10} X_{b}^{n} } \right) \cdot real\left( {X_{b}^{n\beta } } \right) \cdot \frac{{{\text{d}}l}}{{{\text{d}}z}}\, for\, X_{n}^{b} > 0$$

Please note when $X_{b}^{n} = X$ is negative and *α* is a rational decimal number, the resulting $X^{\alpha }$ becomes a complex number, in that case, we will only use the *real* part of the complex number. The same is the case with $\log_{10} X$ and $X^{\beta }$. Also, the absolute values of *α* or *β* are used in Eqs. (2), (3) and (4) for getting positive exponents.

Step 2: z = tanh(y) or in matrix form as:

5 $$Z_{l} = real\left[ {\begin{array}{*{20}c} {\tanh \;(Y_{1}^{1} )} & \cdots & {\tanh \;(Y_{1}^{b} )} \\ \vdots & \ddots & \vdots \\ {\tanh \;(Y_{n}^{1} )} & \cdots & {\tanh \;(Y_{n}^{b} )} \\ \end{array} } \right] = \left[ {\begin{array}{*{20}c} {Z_{1}^{1} } & \cdots & {Z_{1}^{b} } \\ \vdots & \ddots & \vdots \\ {Z_{n}^{1} } & \cdots & {Z_{n}^{b} } \\ \end{array} } \right]$$

Here since all the operations are an element-wise matrix operation, the matrix calculated using (2) is passed to matrix calculation as in (5), then the output matrix $Z_{l}$ of layer $l$ is passed into the pooling layer. For the layer loss $\frac{{{\text{d}}l}}{{{\text{d}}X}}$, first the derivative of $Y_{l}$ with respect to (w.r.t) $X_{l}$ is calculated using Eq. (6), so that the output $Y^{\prime}$ dimension matches exactly the dimension of the layer input i.e., $X_{l}$.

6 $$\begin{aligned} Y^{\prime} & = \frac{{{\text{d}}Y_{l} }}{{{\text{d}}X_{l} }} = 0.1 \times \alpha \cdot real\left( {X^{\alpha - 1} } \right) \,for\, X < 0 \\ & = 1.1 \times \beta \cdot real\left( {X^{\beta - 1} } \right) \,for\, X \ge 0 \\ \end{aligned}$$

Then, the overall gradient loss $\frac{{{\text{d}}l}}{{{\text{d}}X}}$ is calculated through the output of this layer as the derivative of $Z_{l}$ w.r.t $Y^{\prime}$, which is backpropagated to the former layers using Eq. (7).

7 $$\frac{{{\text{d}}l}}{{{\text{d}}X}} = \frac{{{\text{d}}Z_{l} }}{{{\text{d}}Y^{\prime}}} \cdot \frac{{{\text{d}}l}}{{{\text{d}}Z}} = \frac{{{\text{d}}\tanh \left( {Y^{\prime}} \right)}}{{{\text{d}}Y^{\prime}}} \cdot \frac{{{\text{d}}l}}{{{\text{d}}Z}} = {\text{sech}}^{2} \left( {Y^{\prime}} \right) \cdot \frac{{{\text{d}}l}}{{{\text{d}}Z}}$$

Here, $\frac{{{\text{d}}l}}{{{\text{d}}Z}}$ is the loss back-propagated from the deeper layers. Since z = tanh(y) is used as a squashing function, the final output value of the layer is non-uniformly scaled before passing out to the next layer resulting in z being a non-symmetric function centered at zero. This is shown in Fig. 2c and d, where d(proposed-SGT) shows the plot for the final output of the first-order derivative of the proposed function. For condition with exponents *α* and *β* both being 1, the activation layer behaves like tanh in the positive part and leaky ReLU in the negative part, whereas for the case of derivative, the first-order derivative is a constant so behaves exactly like Leaky ReLU with output constant 0.3592 and 0.99006 for positive and negative part respectively. Such behavior was observed in few filters with *β*(positive) > *α*(negative) as in the 18th filter which seems to be constant output as in two different filters non-lineared at 0. However, since both *α* and *β* are channel-wise learnable parameters, the value is not the same for all the channels (please see Fig. 8). The final value of *α* and *β* were examined to be between − 0.2 and 1.3, and rarely the identical value. Regarding our experiment, in most of the filters, the values of both *α* and *β* were a positive rational number with decimals, and *β* being greater than α in the majority case. More discussion on this is done in the discussion section. In the case with both *β*(positive) > *α*(positive), follows the graph as in 31st filter (please see graph Fig. 2d) where the gradients value for positive *x* gradually keeps on decreasing with the value of *x*, however, the rate of decrease is lower than the tanh derivate. This helps to prevent gradients values from becoming infinitely small, whereas in the negative derivative part the value is almost constant and fairly equals to become 1, for all cases. So, the network becomes less prone to the vanishing gradient or exploding gradient. It is to note that when the input *X*, *α*, *β* becomes 0, it causes an indeterminate form as Sech (0) = ∞ also log (0) = ∞ in this case, we simply replace the value of the parameters as 0.001 to continue training. Few values were recorded undefined still after the convergence (please see Fig. 9), however, they can be ignored.

For training the network and optimizing the parameters we used the Adam^39^ optimization technique. It is a first-order gradient-based optimization algorithm to update parameters until it reaches convergence. The learnable parameter ($w_{t}$) (weights/bias/defined terms like *α* and *β*) during $t{\text{th}}$ iteration is updated using Adam optimization as follow:

8 $$w_{t + 1} = w_{t} - \frac{{am_{t} }}{{\sqrt {v_{t} } + \varepsilon }}$$

where $a$ is the learning rate constant-value kept at 0.001 in our case, $\varepsilon$ is a very small regularization constant value (10^−8^) used as offset to keep a non-zero denominator. An element-wise moving average of parameters gradients ($m_{t}$) and its squared value ($v_{t}$) keeps on being updated as in Eqs. (9) and (10), where $b_{1}$ and $b_{2}$ are decay rates for $m_{t}$ and $v_{t}$ kept at 0.9 and 0.990 respectively.

9 $$m_{t} = b_{1} m_{t - 1} + \left( {1 - b_{1} } \right)\nabla E\left( {w_{t} } \right)$$

10 $$v_{t} = b_{2} v_{t - 1} + \left( {1 - b_{2} } \right)\left[ {\nabla E\left( {w_{t} } \right)} \right]^{2}$$

Here $\nabla E\left( {w_{t} } \right)$ represents the first-order derivative of loss ($E$) for the parameter $w_{t}$, which is the cross-entropy loss i.e.

11 $$loss \left( E \right) = - \frac{1}{N}\mathop \sum \limits_{n = 1}^{N} \mathop \sum \limits_{i = 1}^{K} t_{ni} \ln \left( {y_{ni} } \right)$$

where for $N$ is the total numbers of training samples with $K$ mutually exclusive labels and $t_{ni}$ is targeted output, and $y_{ni}$ is the predicted value with its natural log ($\ln$) calculated for $n$th sample belonging to $i$th class.

## CNN model and methodology

The performance evaluation of the proposed function was done with the classification of three cohorts of MRIs clinically categorized as AD, CN, and MCI obtained from the ADNI website^40^. The demographic detail of the used MRIs is shown in Table 1. Multiple scans from the same patients with different gradient wrapping and scale correction techniques were used to add heterogeneity and increase the number of experiment samples^41^. The detailed architecture used in the analysis is shown in Table 2. The total dataset was divided into three parts viz train, validation, and test set in the ratio of 5:2:3 so that 495 MRIs were used in training, 197 MRIs for validation, and 296 MRIs were separated for testing the trained models.

**Table 1:** Participants' demographics and MRI counts.

Dataset properties	AD participants	CN participants	MCI participants
Male/female	29/36	22/38	54/33
Mean age	73.55/75.43	75.57/74.43	77.06/72.41
Total number of Participant	65	60	87
Number of MRI scans	209	305	474

**Table 2:** CNN baseline architecture used to train and classify the MRI 3D scans.

Layer number	Layer name	Layer description	Output size	Number of learnable parameters
1	Image input	64 × 64 × 64 × 1 images with 'zero-center' normalization	64 × 64 × 64 × 1	0
2	Convolution	64 3 × 3 × 3 × 1 convolutions with stride [1 1 1] and padding 'same'	64 × 64 × 64 × 64	Weights = 1728 Bias = 64
3	Batch Normalization	Batch normalization with 64 channels	64 × 64 × 64 × 64	Offset = 64, Scale = 64
4	layer_gamma3d or ReLU/Leaky-ReLU/tanh	Proposed SGT function with 2 learnable parameters for 64 channels	64 × 64 × 64 × 64	*α* = 64, *β* = 64 or 0
5	3-D Max Pooling	2 × 2 × 2 max pooling with stride [1 1 1] and padding [0 0 0; 0 0 0]	63 × 63 × 63 × 64	0
6	Convolution	64 5 × 5 × 5 × 64 convolutions with stride [1 1 1] and padding 'same'	63 × 63 × 63 × 64	Weights = 512 K Bias = 64
7	Batch Normalization	Batch normalization with 64 channels	63 × 63 × 63 × 64	Offset = 64, Scale = 64
8	layer_gamma3d or ReLU/Leaky-ReLU/tanh	Proposed SGT function with 2 learnable parameters for 64 channels	63 × 63 × 63 × 64	*α* = 64, *β* = 64 or 0
9	3-D Max Pooling	2 × 2 × 2 max pooling with stride [2 2 2] and padding [0 0 0; 0 0 0]	31 × 31 × 31 × 64	0
10	Convolution	64 7 × 7 × 7 × 64 convolutions with stride [1 1 1] and padding 'same'	31 × 31 × 31 × 64	Weights = 1.404 M Bias = 64
11	Batch Normalization	Batch normalization with 64 channels	31 × 31 × 31 × 64	Offset = 64, Scale = 64
12	layer_gamma3d or ReLU/Leaky-ReLU/tanh	Proposed SGT function with 2 learnable parameters for 64 channels	31 × 31 × 31 × 64	*α* = 64, *β* = 64 or 0
13	3-D Max Pooling	2 × 2 × 2 max pooling with stride [3 3 3] and padding [0 0 0; 0 0 0]	10 × 10 × 10 × 64	0
14	Convolution	64 9 × 9 × 9 × 64 convolutions with stride [1 1 1] and padding 'same'	10 × 10 × 10 × 64	Weights = 2.985 M Bias = 64
15	Batch Normalization	Batch normalization with 64 channels	10 × 10 × 10 × 64	Offset = 64, Scale = 64
16	layer_gamma3d or ReLU/Leaky-ReLU/tanh	Proposed SGT function with 2 learnable parameters for 64 channels	10 × 10 × 10 × 64	*α* = 64, *β* = 64 or 0
17	3-D Max Pooling	2 × 2 × 2 max pooling with stride [4 4 4] and padding [0 0 0; 0 0 0]	3 × 3 × 3 × 64	0
18	Fully Connected	1728 fully connected layer	1 × 1 × 1 × 1728	Weights = 2.98 M Bias = 1728
19	Dropout	50% dropout	1 × 1 × 1 × 1728	0
20	Fully Connected	3 fully connected layer	1 × 1 × 1 × 3	Weights = 5.18 K Bias = 3
21	SoftMax	SoftMax function	1 × 1 × 1 × 3	0
22	Classification Output	Cross-entropy with 'AD', 'CN' and 'MCI' labels	1 × 1 × 1 × 3	0

Here, while analyzing the performances of different activation functions, layers containing SGT functions i.e., layer_gamma3d are replaced with other existing standard activation functions. Weights and bias values for convolution and FCL were initialized using the ‘Glorot’ initialization technique and for the proposed SGT layer, *α* and *β* values were randomly initialized between 0 and 1. The initial learning rate was set at 0.001 with learn drop factor of 0.95 after every 10 epochs and fully trained up to 80 epochs.

**Figure 3:**
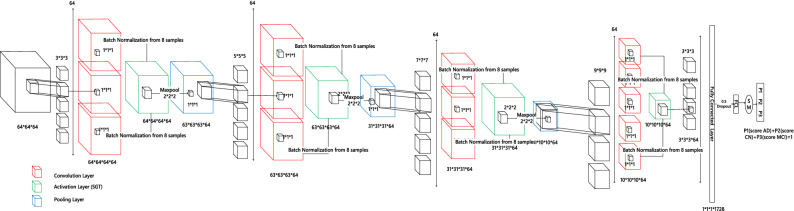
CNN architecture applied for MRI classification using SGT activation.

**Figure 4:**
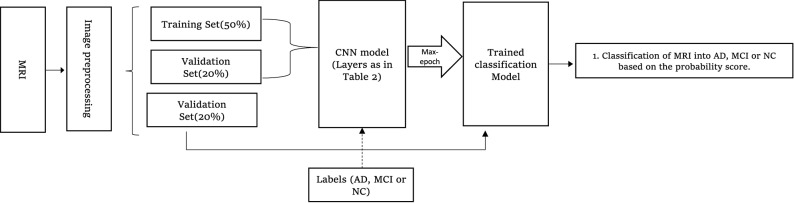
Block diagram for proposed method.

The used CNN architecture is shown in pictorial representation as in Fig. 3, whereas the details of all the layers and number of parameter is shown in Table 2. The used CNN model is based on our previous work^38^, with reduced Fully connected layer to overcome overfitting. As shown in Fig. 3, we can see the size of convolutional kernel keeps on increasing from 3 × 3 × 3 to 9 × 9 × 9 until reaching the final activation size of 3 × 3 × 3. Hence it is called a diverging network ‘divNet’. The proposed SGT layer (green cubes) replaces the ReLU activation originally used in the CNN model. Similarly Fig. 4 represents the whole classification process. The MRI scan in NIfTI (Neuroimaging Informatics Technology Initiative) format is inputted to the CNN after a minimal image preprocessing step to resize into 64 × 64 × 64. Then follows the convolution, normalization, activation, and pooling process in a sequential manner to obtained down sampled feature vector. This encoding process repeats up to the FCL followed by a dropout layer. Finally, a SoftMax layer to output probabilities score, which is used for the loss calculation. The used loss function is cross-entropy, for multiclass classification. Here the major focus in done in the activation layers, its input and output analysis (via histogram) and the behavior of activation function with different learnt value of the learnable parameters.

All experiments were conducted using MATLAB R2022a academic software on Windows 10 OS. Network models were trained on NVIDIA GeForce RTX 3090 GPU with 24 GB dedicated memory and tested in Intel® Core™ i9-10900 K CPU @ 3.70 GHz with 32 GB of memory. The trained mat file will be provided to researchers upon request to the authors.

## Classification performance

**Figure 5:**
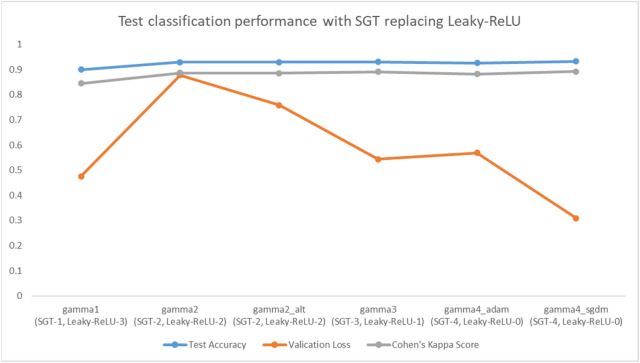
Controlled experiment result with proposed SGT layer gradually replacing Leaky-ReLU layer. Here gamma_X represents the SGT layer where X is the number of SGT layer. In total, we had 4 activation layer replaced in the final experiment. The result keeps on improving with increasing number of SGT layers.

**Figure 6:**
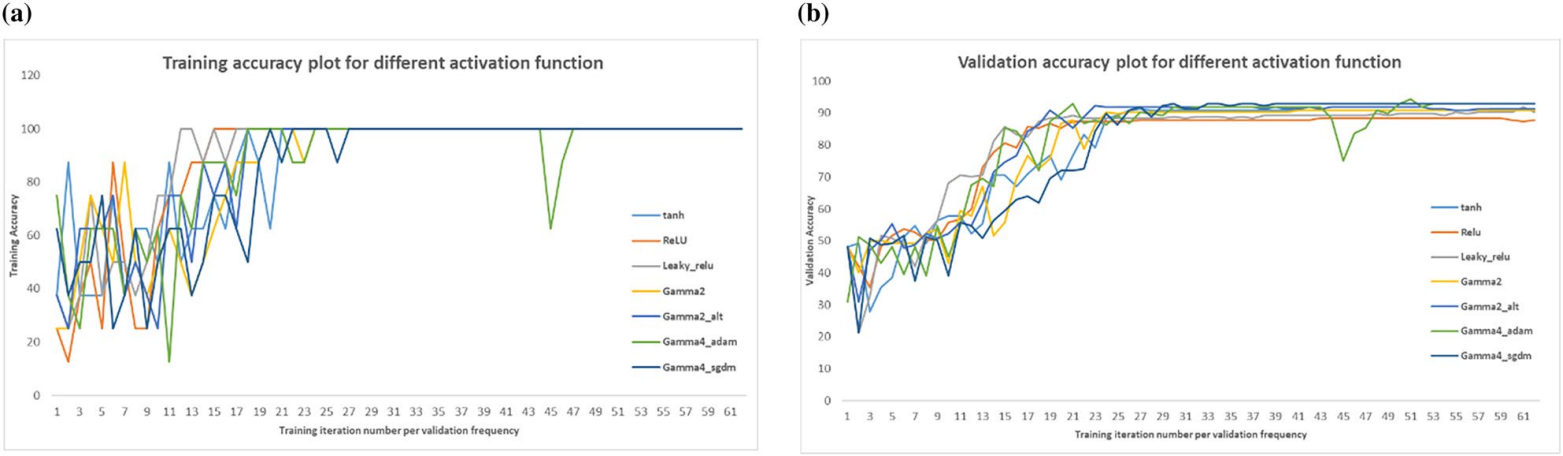
(**a**) Training accuracy plot for MRI classification using baseline CNN models with different activation functions. (**b**) Validation accuracy plot for MRI classification using baseline CNN models with different activation functions.

From Table 3 and Fig. 5, it is observed that all the version of the network using SGT activation (i.e., gamma2, gamma2_alt, gamma4_adam, gamma4_sgdm) has higher validation accuracy than the other activation schemes. These classification performance parameters measure the reliability and correctness of the work, e.g., accuracy measures the number of correct prediction against the true predictions whereas precision measures how close the measured valued are to the true values. Similarly, Cohen’s kappa score is like accuracy except that it is more robust and measures how much better the model is performing over the performance of a model that randomly predicts according to the frequency of each class, best suited for multiclass imbalanced dataset (please see "Appendix" for all formulas). Additionally, to investigate the effect of SGT function precisely, few controlled experiments were performed with gradual replacement of standard activation layer with proposed one. The result of this ablation experiment is shown in Fig. 5, where final test accuracy, final validation loss and Cohen’s kappa score are reported. Here gamma2 means the first two activations are SGT and other ReLU, gamma2_alt means first and third is SGT and other ReLU, gamma4_adam uses all four activation layers as SGT with Adam optimizer while gamma4_sgdm also uses four SGT activation layers but the optimizer is Stochastic Gradient Descent with Momentum (SGDM). The validation set is the test set used during training to calculate the accuracy of prediction at different epochs, hence it helps to know how well the network is learning. Figure 6b shows the validation accuracy calculated at different epochs along with its training accuracy in Fig. 6a. It can be clearly noticed that the SGT activated network (gamma4_sgdm, gamma4_adam) reaches higher validation accuracy than other activation schemes in the final stages of training. The final validation accuracy reported in Table 3 is the accuracy on the validation set at the 80^th^ epoch or the final epoch. Similarly, the test set is the set that is completely unseen for the trained model and the higher performance in the test set means the network is well generalized and has good performance for unseen data. To get an unbiased result, the experimental environment along with all the hyperparameters and participating MRIs were always kept identical for all networks irrespective of the choice of activation functions. During test set classification, Leaky-ReLU performed the best with around 0.5% higher test accuracy than that of gamm4_sgdm. Still, the test accuracy of all SGT activated networks was higher than the ReLU and tanh by 2% and 1% respectively, which indicates that the proposed SGT activation scheme outperforms the traditional ReLU activation by a clear margin. Table 4 shows a comparison of our result with some recent works.

**Table 3:** Results for multi-class MRI classification using CNN architecture as in Table 2.

Type	Name	Final validation accuracy (%)	Test accuracy (%)	Final validation Loss	Cohen’s kappa	Precision (class-wise [AD CN MCI])	Predicted confusion matrix	True confusion matrix
Standard Activation Functions	tanh	90.86	92.57	0.5338	0.897	[0.8889 0.9120 0.9507]	56 1 6 0 83 8 1 6 135	63 0 0 0 91 0 0 0 142
	ReLU	87.81	91.22	1.0425	0.860	[0.9048 0.9011 0.9225]	57 3 3 1 82 8 1 10 131	
	Leaky-ReLU	90.35	**93.92**	0.8201	**0.902**	[0.9206 0.9121 0.9648]	58 2 3 1 83 7 1 4 137	
	Swish	90.35	92.57	0.6022	0.881	[0.9048 0.8901 0.9577]	57 2 4 2 81 8 0 6 136	
SGT Function	gamma4_adam	92.89	92.57	0.5638	0.881	[0.8730 0.9451 0.9366]	55 6 2 0 86 5 1 8 133	
	gamma4_sgdm	**92.89**	93.24	**0.3086**	0.892	[0.9048 0.9121 0.9577]	57 2 4 1 83 7 1 5 136	

Bold result in the table content represents the best case.

**Table 4:** Comparison with other works in MRI classification.

Authors	Methods	Number of MRI scans	Activation function	Testing accuracy (%)
Gupta et al.^32^	Sparse Auto encoder (SAE) based CNN	CN:1278 AD: 755 MCI: 2282	Sigmoid	94.74
Payan et al.^25^	SAE patch-based 3D CNN	CN:755 AD:755 MCI:755	Sigmoid	89.47
Hosseini-Asl et al.^24^	DSA-3D-CNN	CN:70, AD: 70	Sigmoid and ReLU	97.60
Oh et al.^26^	Inception auto encoder-based 3D CNN	NC:230 AD:198 sMCI: 101	ReLU	84.5
Proposed method	Diverging CNN	CN:305 AD:209 MCI:474	SGT	93.24

Please note the difference in number of MRI scans.

## Discussion and analysis

### Histogram analysis and asymmetric distribution

Weights of each layer’s input ($X_{l}$) or output ($Z_{l}$) as in Eqs. (2) or (5) is plotted against its frequency in the histograms. The normalized output values from BN are zero-mean with almost normal distribution, so it is not a good idea to throw away all the negative valued parameters/weights using activation functions like ReLU or sigmoid^19^. Though the flow of gradient is positive in ReLU, if a bunch of the weights is negative it causes dead ReLU with ‘zero’ derivative for negative weights, hence not every time ReLU is a wise choice. In cases like MRI, mostly with black background (low pixel value), it is better to use alternative activation function like Leaky-ReLU, GELU, SELU that provides non-zero gradients for negative weights ensuring the flow of gradient loss.

**Figure 7:**
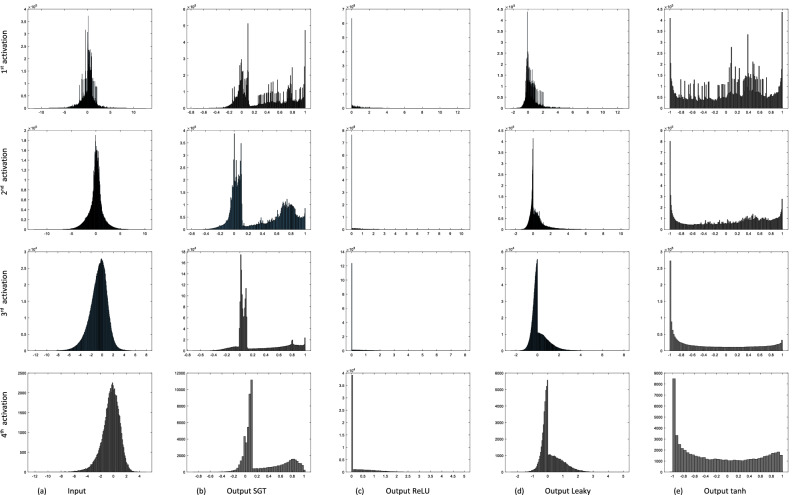
Histogram of the input features against output using various activation functions for a single MRI input plotted for different layers i.e., 4, 8, 12, and 16 (please see Table 2 for layers). Here, the histograms are combinedly produced using all the data values from 64 filters/channels. Generally, the combined histogram of all channels is similar to the single histogram of each channel (please see Fig. 7_app for comparing the histogram plot of 19th filter out of 64 filters for same input MRI in "Appendix" section).

**Figure 8:**
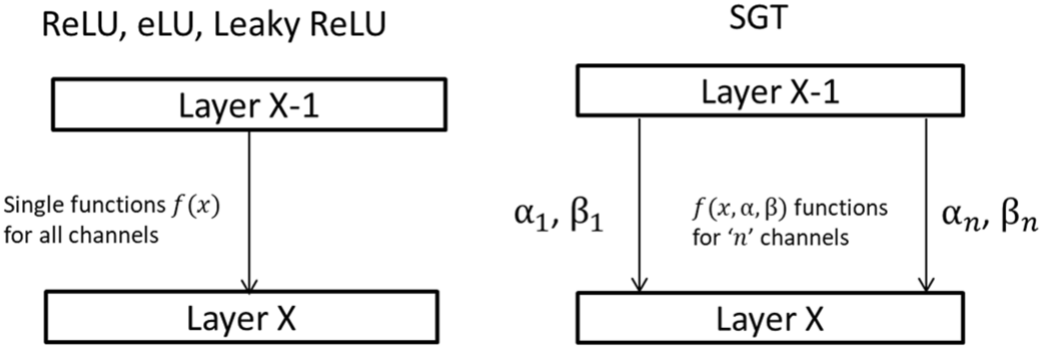
Conventional activation functions work in a constant way to all inputs whereas the proposed SGT function works differently for the different channels because of altering values of parameters $\alpha_{n}$, $\beta_{n}$ within the layers channel in respect to Eq. (2).

Figure 7 shows the input and output histogram plots through the SGT layer in comparison to ReLU versions. Here, please note that the input to the activation layer is the output from the batch normalization and the output of the activation layer is the input to the pooling layer. In Fig. 7a, the input histogram of all activation layers has an almost symmetrical distribution which means most of the image pixel lies in the grey region after BN. Our goal of gamma correction is to reduce this grey zone and make the distinction between white (bright) and black (dark) regions. If we look at Fig. 7b, the mid-grey region is very few in the case of output from the proposed SGT layer, whereas the output with ReLU Fig. 7c, has very high zeros and leaky-ReLU Fig. 7d output still seems centered at zero, hence the clear skewness is seen in positive part. While the SGT layers’ output data are decentralized in opposite edge regions unlike BN, and it seems like the combination of the output of tanh and Leaky-ReLU histogram. Additionally, this asymmetric feature distribution in the SGT layer supports the classification task due to the higher variance between the edge regions (Fig. 8).

### Channel wise activation

**Figure 9:**
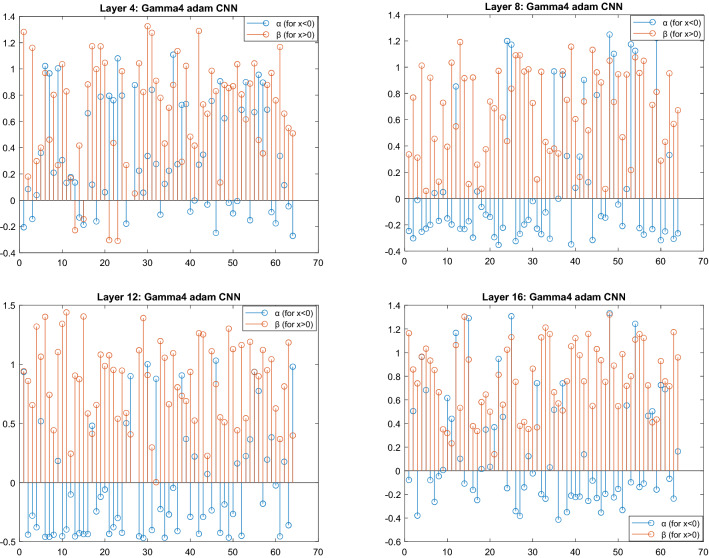
Pictorial representation of *α* and *β* values for a trained model at different layers for gamma4_adam network using Adam optimization. Here *α* and *β* are channel-wise learnable parameters in SGT layers, each corresponding to 64 channels.

We were particularly interested to see what value the SGT (layer_gamma3d in Table 2) parameters would learn at different activation layers. The stem plot for *α* and *β* values from all activation layers as in Fig. 9 shows that for the first SGT activation layer (i.e., Layer 4) the values for *α* and *β* were mostly positive and only a few remained negative, also there were more *β* with value > 1 than *α*. The range for the value of *α* and *β* lied between − 0.4 and 1.4. Interestingly in the intermediate activation layers (i.e., Layer 8, 12) and the final activation layer (i.e., Layer 16) none of the values for *β* remained negative while the values for *α* in most channels remained negative. This might imply that for feature value *x* > *0*, required positive gamma correction, and for negative feature value *x* < *0*, required negative gamma correction in the intermediate layer. In a more general statement, the gamma activation made brighter pixels look brighter and darker pixels look darker, which resulted in a more distinct intensity profile.

### Analyzing weights and bias in the final fully connected layer

**Figure 10:**
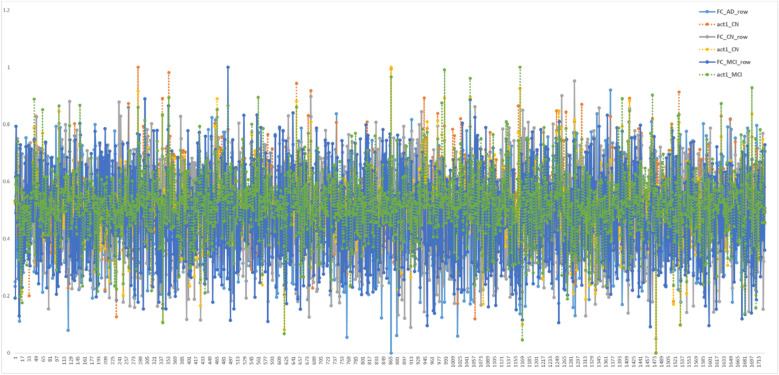
Final FCL 1728 weights plots of trained gamma4_adam network corresponding to each class label. Here FC_AD_row represents the final weights of the layer from the fully trained gamma4_adam network belonging to the AD class, similarly, FC_CN_row and FC_MCI_row represent for CN and MCI categories respectively. While the plots of act1_AD are the weights calculated for a typical AD categorized MRI, obtained using the trained model during the testing phase. So, are the weights calculated as act1_CN and act_MCI for a CN and MCI categorized MRI during testing respectively. This plot is to show how closely the test sample (act1_xx) follows its parent class characteristics (FC_xx_row). Furthermore, to evaluate this characteristic a correlation table is calculated as in Table 5, where it is very clear that the test sample weights (act1_xx) have the highest correlation with its parent class (FC_xx_row) where xx represents the same class for both sample and parent. The same class high correlation between FC_xx_row and act_xx shows that the network is learning class-wise property precisely.

FCL represents an MLP Feedforward network with learnable weights and bias but mostly without activation function when used in CNN^13,14^. In FCL all inputs are mapped to output unlike the convolutional layers which are used as a patch-based feature extractor, therefore weights and bias in FCL are highly responsible for predicting the result, and the weights themselves suggest which input has more effect (or gain) on output. Thus, the weight distribution pattern of FCL might indicate how a network behaves during the test phase. To interpret this, we plotted all trained weights of the final FCL (Input nodes = 1728, output nodes = 3, connection = 5184) for all 3 classes as shown in Fig. 10. Later the correlation matrix is calculated as in Table 5, which shows a sample MRI’s features (or weights) calculated from the FC layer is closely correlated with its parent class. For instance, the test sample CN MRI’s FC weights i.e., act1_CN has correlation value [0.143417277 0.24265146 − 0.009627914] with the trained network corresponding layer weights [FC_AD_row FC_CN_row FC_MCI_row]. So, the highest correlation value is 0.24265146 for FC_CN_row implies, the MRI test sample has a higher affinity for ‘CN’ class weights during classification besides, it supports the logic behind why the network predicts the test sample label as ‘CN’.

**Table 5:**
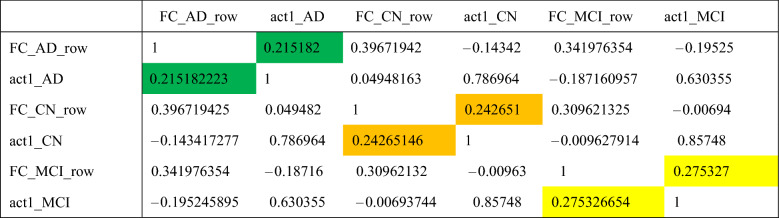
Correlation matrix for weights as shown in Fig. 10.

The colored ones are the highest measured value for the sample-parent pair, higher being better.

After weights analysis, we were also interested to analyze the bias value. So, the idea is to check how much network is biased to each class via calculated bias in the final FCL layer. The obtained bias value is from the last FCL, which goes into SoftMax for probability calculation. We know weights in the network directly influence the output value for input, whereas bias works as a regularization constant to make non-zero output when input/weights are zero and don’t have a successive layer-wise influence on the output. Although it is difficult to exactly interpret the bias value theoretically, we assume the bias values close to each other cohort, can correlate how each other is numerically related. E.g., for tanh trained CNN the obtained bias value is [AD CN MCI] = [− 0.006021075 0.000316184 0.004943716], which means that AD (with negative value) is closely related to CN (small positive), being the difference of value between AD and CN greater than AD and MCI, which is against the general assumption that AD is closely related to MCI, both being a dementia condition. This might also indicate that the tanh network can easily differentiate between AD and MCI rather than AD and CN, which is not what it should be, the same is the case with Leaky ReLU. Surprisingly, this might be supportive for the classification task, as higher difference in bias would make the network easier to calculate the class-wise probabilities scores. On the contrary, the proposed SGT networks (gamma4_adam and gamma4_sgdm) have a larger difference between AD and CN bias values, one being positive and the other being negative. While MCI is nearly 0 indicating a moderate status between AD and CN. The lower difference in MCI and CN bias values in the gamma4_adam network might suggest a higher difficulty in classification and generalization between CN and MCI, which supports the real scenario.

Moreover, to analyze the performance of our model we have evaluated our model classification performance using t-SNE 3D projection. Figure 12 represents the 3D t-SNE projection for visualization of reduced features from the final FCL. The features into the FCL are originally from multiple channels later reduced into a single channel, so are considered flattened features. However, each MRI’s flatten feature needs to be reduced to a 2D or 3D dimension for proper visualization. The distinctive clustered distribution in the projection means the network is learning class discriminant properties with good fitness. This can be seen in Fig. 12c, SGT produces better dense class clustering (seen via color) than other ReLU and Leaky-ReLU.

## Conclusion

**Figure 11:**
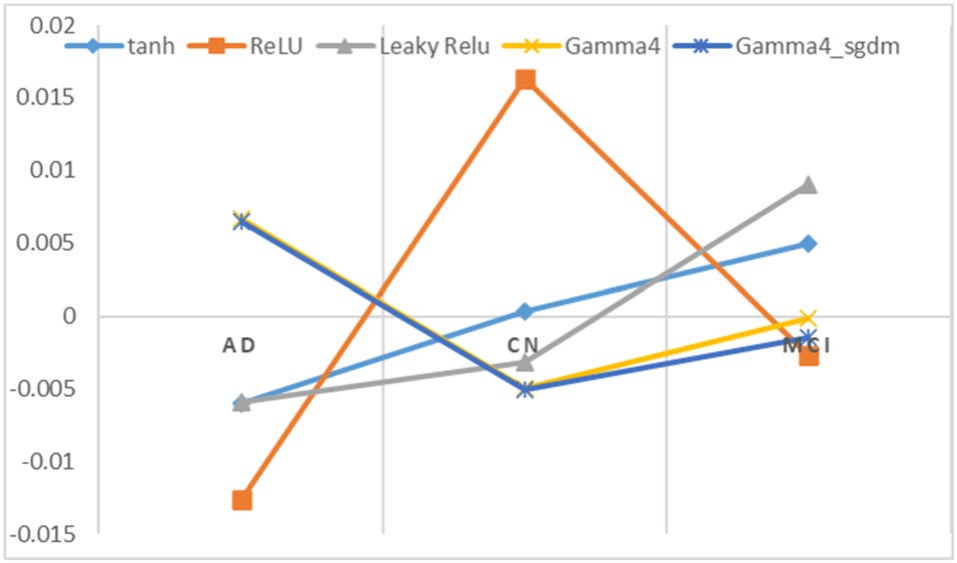
Bias value plot of final FCL layer from the baseline CNN model using different activation functions.

**Figure 12:**
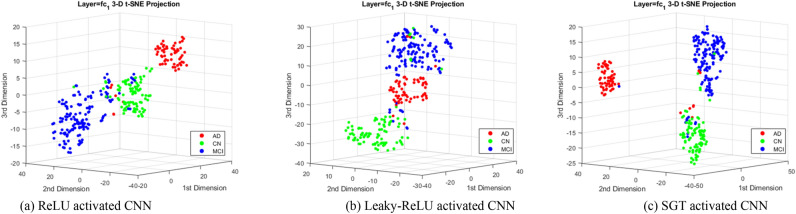
3D projection viewed at the same angle for the test set features reduced from 1728 dimension to 3 using the t-SNE algorithm. Here each color dot represents an MRI scan, hence a total of 296 dots for 296 test MRI. The non-linear feature distribution shows the requirement of complex boundaries for classifications. Here the figure from left to right is obtained as the result of t-SNE distribution using ReLU, Leaky-ReLU, and SGT activation separately in the same baseline 3D CNN model. Please see Fig. 12_app in the "Appendix" section for the 3D t-SNE projection of all individual layers in the gamm4_adam network.

DNN design and hyperparameter selection are task-specific with no single model or function that can work universally for all, however, after all the experiments and analysis we can conclude:

- A novel channel-wise dynamic activation function is introduced with superior performance than standard ReLU and tanh function in 3D CNN for MRI classification.
- We showed that the proposed activation function can diminish the negative gradient loss arising with the negative weights with less likelihood for vanishing or exploding gradient problem and also zero gradient problem unlike dead ReLU (please see derivative plots in Fig. 2c and d) for shallower networks.
- The analysis performed in histograms (Fig. 7), showed negative weights are produced in a quite large measure during convolution and batch normalization operation so, the idea of utilizing negative weights to relatively contribute to the gradient loss proved meaningful with the proposed activation function.
- We tried to explore the pattern of weights and bias in the final FCL and how numerically they might be related (Figs. 11, 12, and Table 5) in regard to the classification task. This might be one of the few attempts in this field as weights can be optimized in numerous approaches but difficult to analyze.
- Since DNN are very prone to overfitting so we cannot be very sure about this. However, under identical training environment (i.e., hyperparameter, model and training material) if there is overfitting, it would affect in both cases (i) using standard activation and (ii) using SGT. Hence, in both case the result will be biased, so that our final goal to compare the SGT characteristic against other activation functions is equally affected by overfitting (if any). And if the use of parametric layer brings some overfitting, we have reported the validation and test accuracy for all cases so, still the result is convincing for SGT.

Our idea is quite simple as well as interesting so we hope, our work could be helpful and meaningful for other researchers working in deep learning. In the future, more modifications are required for superior performance than all other activation functions and to work universally in all kinds of the image dataset.

## Supplementary Information

Supplementary Information.

## Data Availability

Data used in this article are publicly available on Alzheimer’s disease Neuroimaging Initiative (ADNI) database: https://ida.loni.usc.edu accessed on 10 February 2021. All methods were carried out in accordance with relevant guidelines and regulations as stated in the official website http://adni.loni.usc.edu/methods/documents/ accessed on 30 January 2022.
