# Supplementary material for: A novel scaled-gamma-tanh (SGT) activation function in 3D CNN applied for MRI classification
Source: Sci Rep. 2022 Sep 2;12:14978. doi: 10.1038/s41598-022-19020-y (PMC9440075; doi:10.1038/s41598-022-19020-y)
Supplement: Supplementary file 1 — Supplementary Information. [file 41598_2022_19020_MOESM1_ESM.pdf]

Appendix

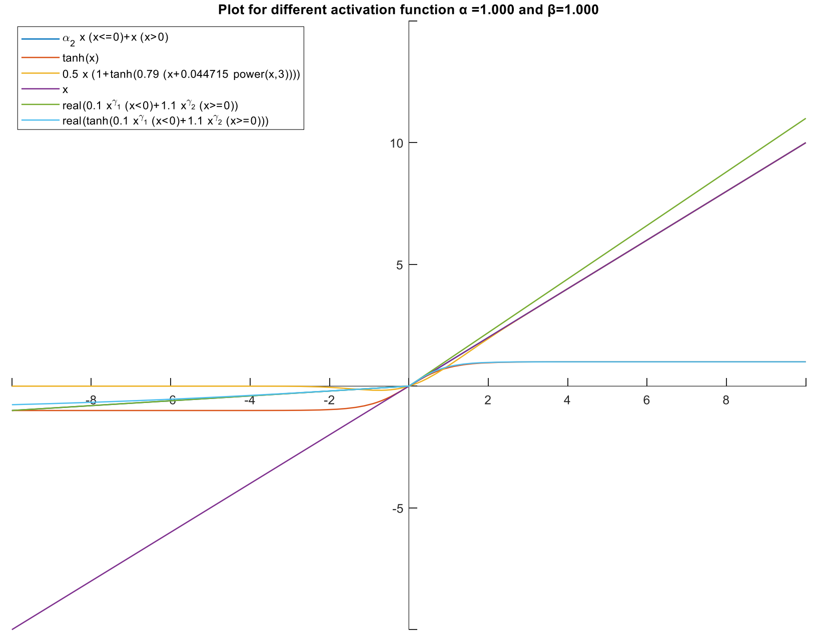

Figure 2(a)_app : Here the last sky blue graph represent the proposed-SGT function.

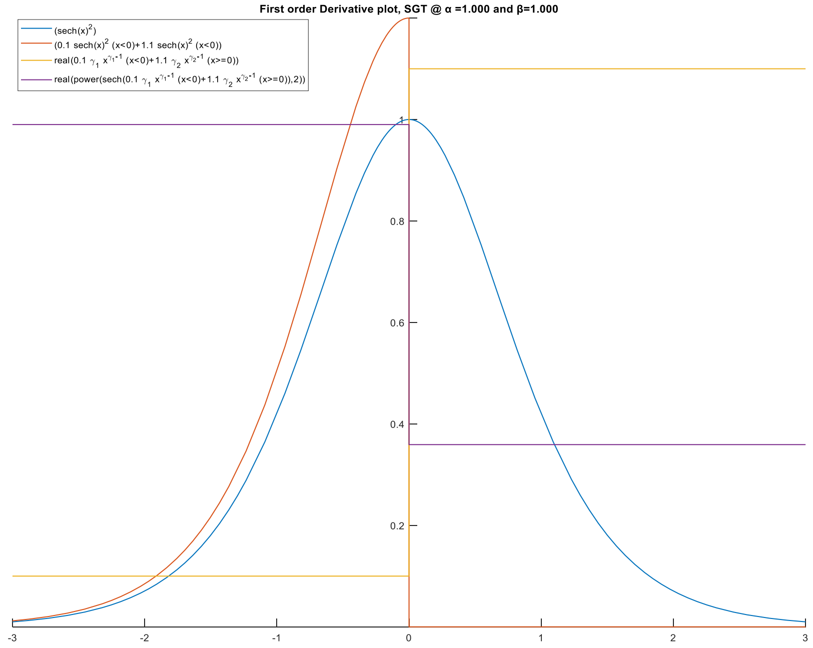

Figure 2(b)_app : Here the last purple curve represents the derivate of proposed-SGT function.

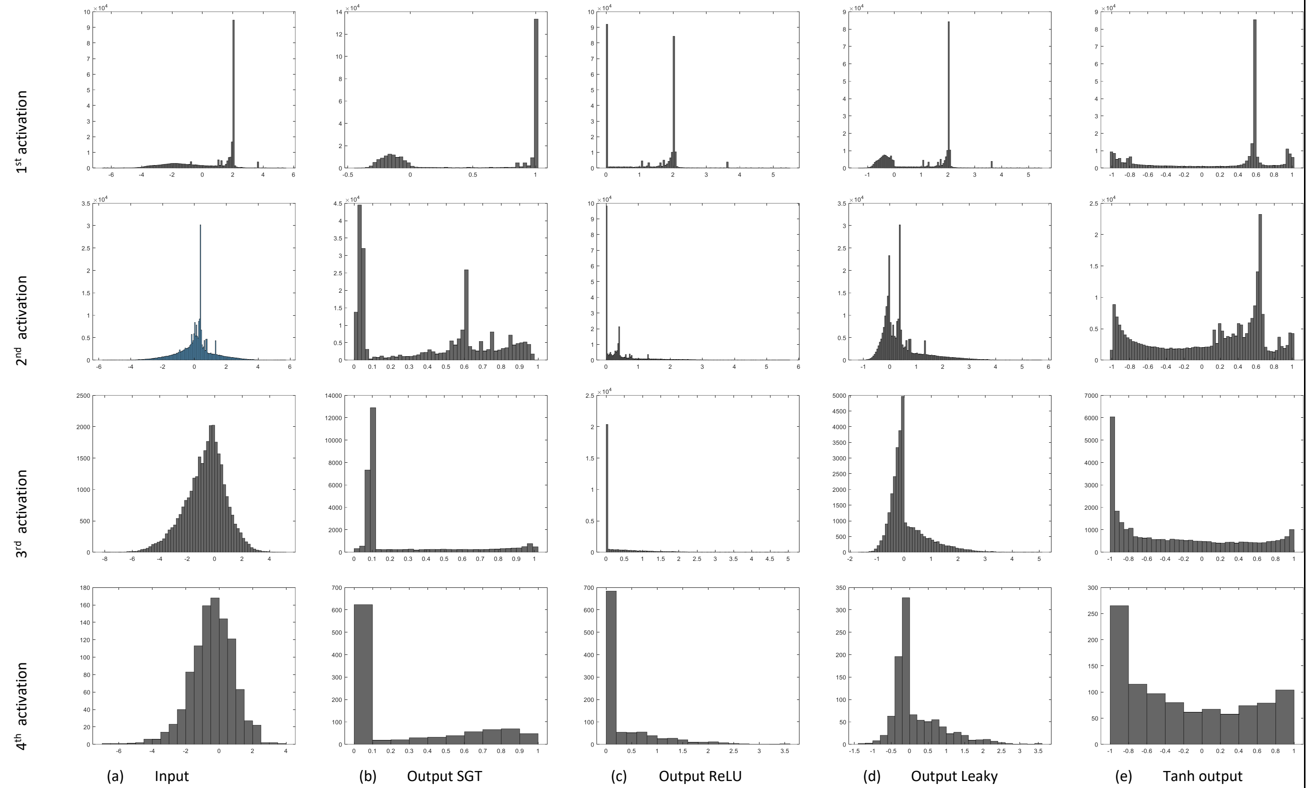

Figure 7_app. Histogram plot of 19th filter out of 64 filters for the input features against output using various activation functions for a single MRI input plotted for different layers i.e 4, 8, 12 and 16 (see Table 2 for layers and Figure 4 for all channels histogram)

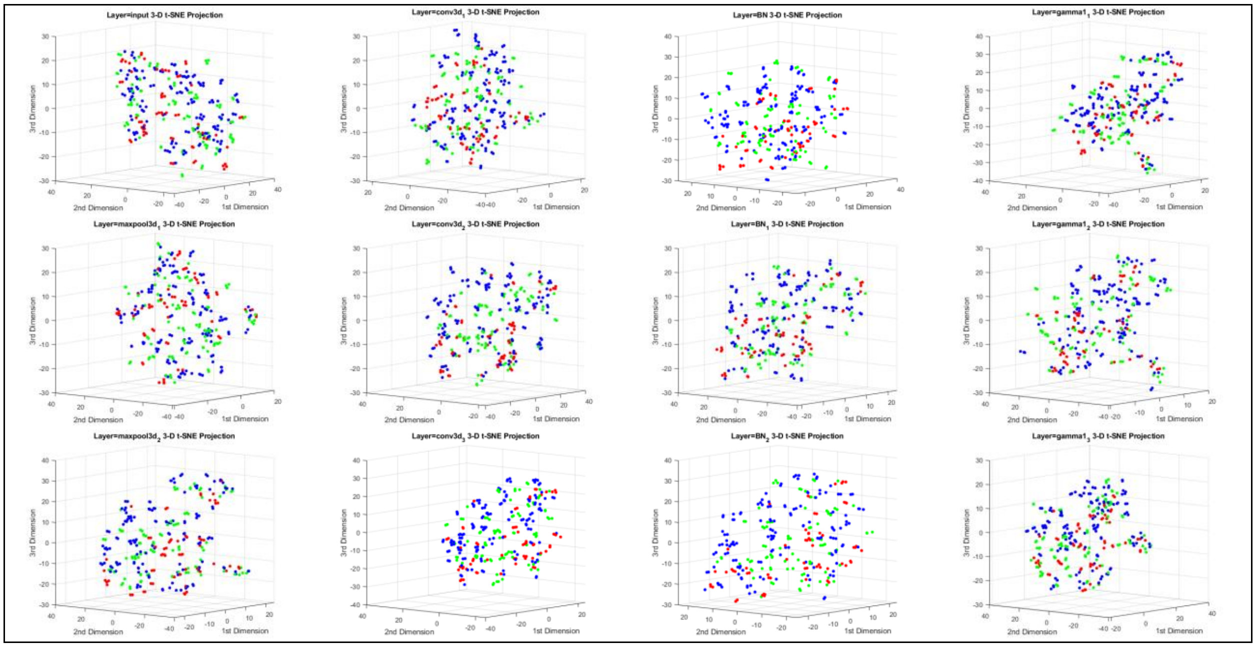

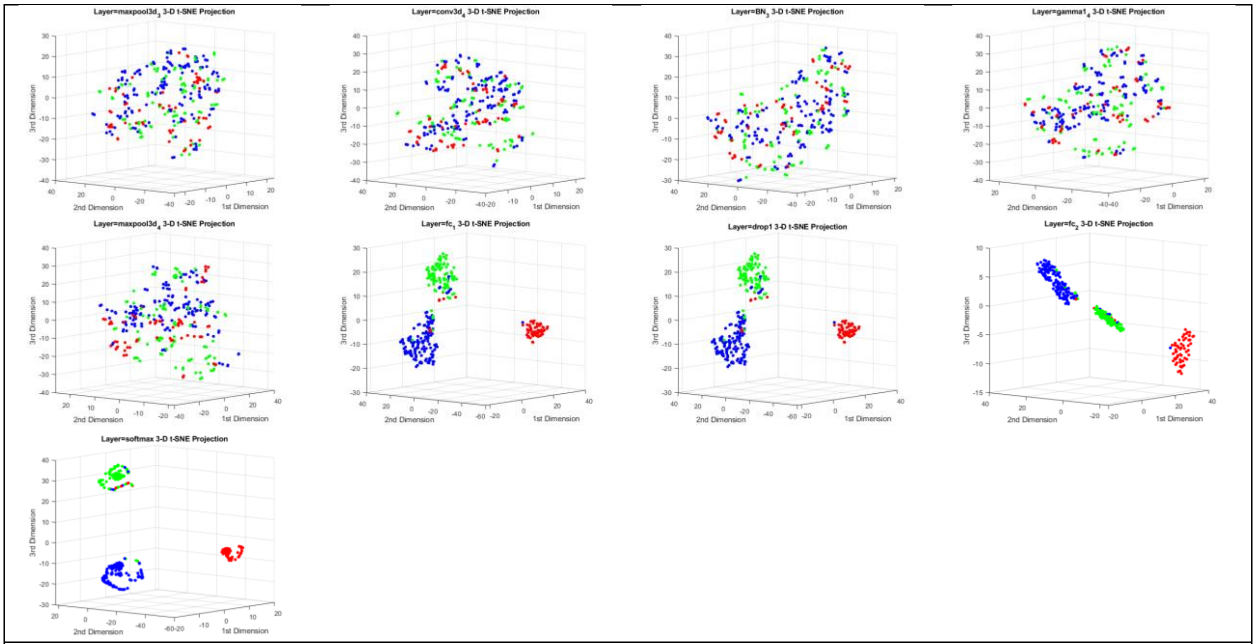

Figure 12_app. 3D t-SNE projection of all individual layers in gamm4 network. Here each red dots represents an AD subject MRI, blue dot represents MCI and green dot represents CN MRI.

Matlab code implementation for `layer_gamma3d` **layer as in Table 2 .**

```
classdef layer_gamma3d < nnet.layer.Layer
    properties(Learnable)
        Alpha
        Beta
    end

    methods
        function layer = layer_gamma3d(numchannel,name)
            layer.Name = name;
            layer.Description = " Proposed SGT layer with" +numchannel + "
channels";
            layer.Alpha = rand([1 1 1 numchannel]);
            layer.Beta = rand([1 1 1 numchannel]);
        end

        function Z = predict(layer, X)
              X(isnan(X)) = 0.1;          %for NaN case
              layer.Alpha(isnan(layer.Alpha))= 0.1;
              layer.Beta(isnan(layer.Beta))= 0.1;
              layer.Beta=abs(layer.Beta);
              layer.Alpha=abs(layer.Alpha);
              X_F= 0.1.*(power(complex(X),complex(layer.Alpha,0)));
              check_X = 1.1*(power(complex(X),complex(layer.Beta,0)));
              X_F(X>0) = check_X(X>0);
              Z = tanh(real(X_F));
        end

        function [dLdX,dLdAlpha,dLdBeta] = backward(layer, X, ~, dLdZ, ~)
```

```
        X(isnan(X))= 0.001;                     %for NaN case
        dLdZ(isnan(dLdZ))= 0.001;
        layer.Alpha(isnan(layer.Alpha))= 0.001;
        layer.Beta(isnan(layer.Beta))= 0.001;
        layer.Beta=abs(layer.Beta);
        layer.Alpha=abs(layer.Alpha);
        X_loss = 0.1.*layer.Alpha.*real(power(complex(X),(layer.Alpha-1)));
        dLdX = power(sech(X_loss),2).*dLdZ;
        X_loss2 = 1.1*layer.Beta.*real(power(complex(X),(layer.Beta-1)));
        check = power(sech(X_loss2),2).*dLdZ;
        dLdX(X>0) = check(X>0);
        dLdAlpha =
0.1.*real((log10(complex(X))).*(real(power(complex(X),complex(layer.Alpha,0))
).*(X<0))).*dLdZ;
        dLdAlpha = sum(dLdAlpha,[1 2 3]);
        dLdAlpha = sum(dLdAlpha,5);
        dLdBeta =
1.1*real((log10(complex(X))).*(real(power(complex(X),complex(layer.Beta,0))).
*(X>0))).*dLdZ;
        dLdBeta = sum(dLdBeta,[1 2 3]);
        dLdBeta = sum(dLdBeta,5);
        end
    end
end
```

Outcome of the diagnostic test	Condition (e.g. Disease) As determined by the Standard of Truth		
	Positive	Negative	Row Total
Positive	TP	FP	TP+FP<br>(Total number of subjects with positive test)
Negative	FN	TN	FN + TN<br>(Total number of subjects with negative test)
Column total	TP+FN<br>(Total number of subjects with given condition)	FP+TN<br>(Total number of subjects without given condition)	N = TP+TN+FP+FN<br>(Total number of subjects in study)

Sensitivity = TP/ (TP + FN) = (Number of true positive assessment)/ (Number of all positive assessment)

Specificity = TN/(TN + FP) = (Number of true negative assessment)/(Number of all negative assessment)

Accuracy = (TN + TP)/(TN+TP+FN+FP) = (Number of correct assessments)/Number of all assessment)

Cohen's Kappa score (***κ***)

$$\kappa = \frac{2 \times (TP \times TN - FN \times FP)}{(TP + FP) \times (FP + TN) + (TP + FN) \times (FN + TN)}$$
